# A Point-of-Care Based on Label-Free Interferometric Optical Detection Method to Evaluate Interferon Gamma (IFN-γ): A Correlation with the ELISA Technique

**DOI:** 10.3390/s20174776

**Published:** 2020-08-24

**Authors:** María Fe Laguna Heras, Yolanda Ramirez, Celia Fernández Martín, Rocío L. Espinosa, Alvaro Lavín, Miguel Holgado

**Affiliations:** 1 Center for Biomedical Technology, Optics, Photonics and Biophotonics Lab., Universidad Politécnica de Madrid, Campus Montegancedo, Pozuelo de Alarcón, 28223 Madrid, Spain; y.ramirez@biod.es (Y.R.); rocio.lopez@ctb.upm.es (R.L.E.); alvaro.lavin@upm.es (A.L.); m.holgado@upm.es (M.H.); 2 Department of Applied Physics and Materials Engineering, Escuela Técnica Superior de Ingenieros Industriales, Universidad Politécnica de Madrid, C/José Gutierrez Abascal 2, 28006 Madrid, Spain; 3 BioOptical Detection SL, Centro de Empresas, Campus Montegancedo, 28223 Madrid, Spain; c.fernandez@biod.es

**Keywords:** point-of-care, label-free detection, interferometric optical detection, interferon gamma, ELISA technique

## Abstract

Interferon-gamma (IFN-γ) is a cytokine associated with inflammatory diseases, virus, infection, etc. The quantification of interferon-gamma concentration levels is studied to relate the immune system response to the progression of disease. In this work, we used a label-free point-of-care device based on the increase relative optical power (IROP) and a biosensor based on photonic transducers called BICELLs (Biophotonic Sensing Cells) to evaluate interferon-gamma concentrations. The BICELLs’ sensing surface size used is 100 μm in diameter. The bioreceptor is attached to the surface by streptavidin-biotin affinity. This label-free IROP-based device can work with a low concentration of reagents and a low sample volume for measurements. Furthermore, this new device was compared with an ELISA technique in the same conditions. A good correlation was achieved between both techniques. This device is easy to use, and it is a cost-effective tool for monitoring an analyte in a disease.

## 1. Introduction

There are millions of people affected by autoimmune diseases in the world. Diseases like lupus, multiple sclerosis, Crohn’s disease, autoimmune hepatitis, rheumatoid arthritis, etc. are autoimmune diseases that affect a lot of people; usually, women are more affected by these disorders [1,2,3,4,5]. The immune system protects the body against invaders (viruses or bacteria). In normal conditions, an immune response cannot be triggered against the cells of one´s own body. However, when immune cells attack the cells that they must protect, an autoimmune disease affects the own body. Cytokines are proteins that mediate the activation and communication of the immune system response. Interferons are secreted as a signal to viruses or pathogenic microorganisms.

Isaacs and Lindenmann discovered a protein called interferon that protects cells in vitro from viral infection [6]. Interferon is a family of proteins with antiviral activity secreted in response to stimuli. There are three types of interferons: type I, type II, and type III. Interferon gamma (IFN-γ) is a type II protein. The protein structure of this interferon is different from type I and type III [7,8,9,10].

Interferon-gamma is a cytokine secreted by T CD4+, CD8+, Tγ/δ, and NK cells [11]. The secretion of IFN-gamma is associated with inflammatory diseases, virus infection, Alzheimer´s disease, Crohn´s disease, cancer, etc. Over the years, quantification of interferon-gamma concentration levels has been studied to relate the immune response to the progression of disease. Laboratory methods like Interferon-Gamma Release Assay (IGRA) are wide used and the IFN-γ concentration is evaluated using techniques, such as Enzyme-Linked Immunosorbent Assay (ELISA) [12], Enzyme-linked immunospot assay (ELISPOT), or reverse transcriptase polymerase chain reaction (RT-PCR) [13]. These techniques are expensive and specialized technicians are needed in the laboratory. There are other techniques, such as field effect transistor-based biosensors (Bio-FETs) [14,15] or Förster Resonance Energy Transfer (FRET) [16], that are very good in IFN-gamma detection, with very low detection limits, but they are expensive in the development of biosensors and the reproducibility is not good. Additionally, aptasensors, for the rapid detection of IFN-gamma at low concentrations, have been developed (in order to picomolar concentrations), but the fabrication of these biosensors is complex and the detection in real samples decreases [17,18,19,20].

Currently, there is a need for cytokine sensors for use in clinical testing, the development of new biomarkers, and diagnosis and treatment of diseases. In this sense, the last works for interferon gamma detection have been based on electrochemical biosensors [15,19,21] and also optical detection [22,23]. One cytokine assay based on the affinity between the cytokine and their specific antibody is usually used in optical mechanisms. In addition, nanomaterials used in optical biosensors show optical properties that show improved performances in the analyses of cytokines. For this reason, the optical label-free biosensors are a kind biosensor that offer advantages, including avoiding the label process, and a simplicity in terms of assay protocols with minimum steps and a low volume of analytes [24,25,26].

Our research group has developed a point-of-care device based on optical label-free detection. The device is a compact and cost-effective point-of-care based on the “increase relative optical power” (IROP) principle and the enhancement in performance and LoD in comparison with standard high-resolution spectrometry [27,28,29,30]. The “increase relative optical power” quantifies the biomolecule accumulation or recognition in one sensing surface. The calculation is based on the reflectivity as a function of the wavenumber and wavelength for the reference interferometer and for the signal interferometric. The interferometric profile of the Fabry-Perot defines one area that it is proportional to the optical power in the situations when the biomolecule is attached on the surface.

Furthermore, the transducers used are based on Fabry-Perot interferometers called BICELLs (Biophotonic Sensing Cells) [31,32,33]. BICELLs are fabricated with two materials with a refractive index that shown an improvement of the optical interferometric profile.

In this work, we demonstrate the capability of the PoC device together with a transducer based on biophotonic sensing cells (BICELLs) to evaluate the concentration of interferon-gamma compared with the ELISA technique, where several steps are required and more reagents are used. The goal of this paper was the use of the PoC device for the evaluation of a cytokine with a low volume of reagents in a label-free detection with minimum biochemical steps.

For this proposal, an interferon-gamma detection experiment was performed on a sensor surface based on the affinity of streptavidin-biotin with the corresponding antibody using a very small volume in each biocell.

In addition, we report a very good correlation between the new technology based on label-free detection and one gold standard laboratory technique, such as the ELISA technique.

## 2. Materials and Methods

### 2.1. BICELLs Fabrication and Materials

**Figure 1:**
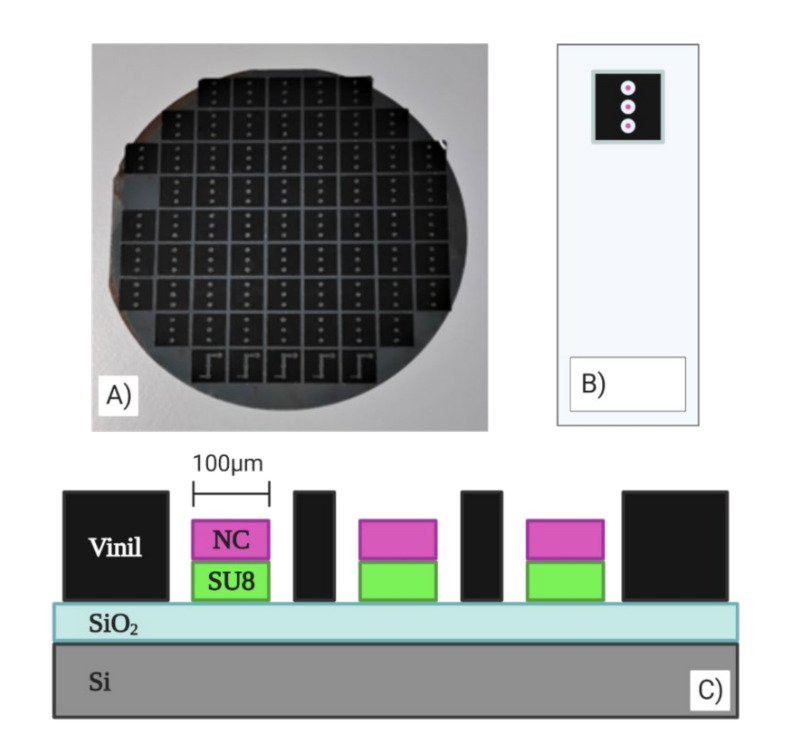
(**A**) Chip fabricated at the wafer level. (**B**) 100-µm BICELLs integrated in chips with a vinyl sticker. (**C**) Transverse view of the transducer.

A compact chip based on BICELLs was fabricated by two interferometric layers, SiO_2_ and SU8 resist (Microchem.), and another thin layer of nitrocellulose (Sigma-Aldrich). The manufacturing was carried out at the wafer level according to previous reported works [33]. The SU8 resist was overlaid on the wafer, after it was exposed to a thermal treatment, and it was cured by optical contact lithography with a mask. Finally, the wafer was developed to remove the remaining resist. The design of the mask allowed us to fabricate a chip with three BICELLs of 100 μm in diameter. The last layer of the biosensor was a nitrocellulose layer spun over the Bicells and exposed by DUV with a quartz mask inversely copied from the mask used in the lithography process. The remaining nitrocellulose was eliminated with a developer AR-600-55 (AllResist GmbH, Strausberg, Germany) diluted 1:4 with propanol. Each chip had three BICELLs to study the repeatability and reproducibility of the sensing surface, as shown in Figure 1.

### 2.2. Point-Of-Car: Optical Read-Out Device

**Figure 2:**
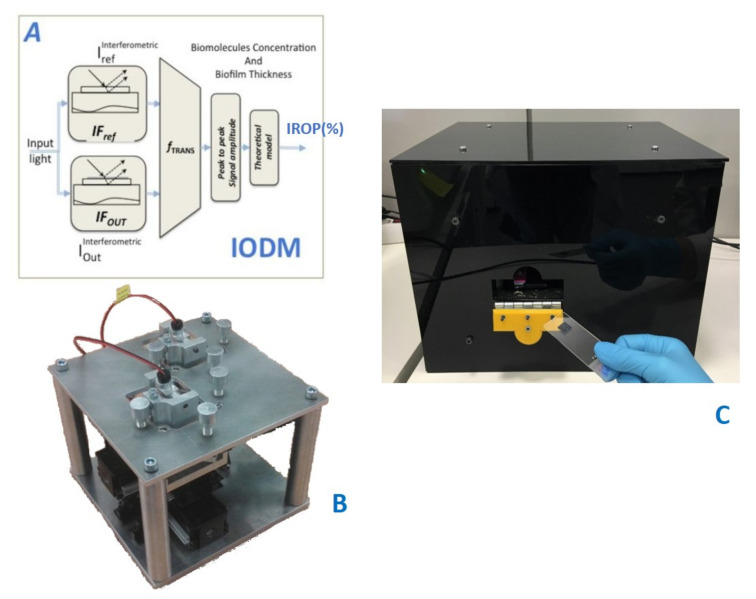
(**A**) Read-out method. From [27] (**B**) Platform Design with optical channels and XY-positioning stage. (**C**) Point-of-care device.

The read-out methodology for this point-of-care device is based on the increase relative optical power (IROP) value, previously introduced in several publications [27,28,30], and defined as the quotient of the optical power of two interferometers: signal (I_Sig_) and reference (I_Ref_) interferometers in a certain optical band, given by the light source employed (Figure 2).

Both interferometers consist of FPIs with different configurations in a way that the reference interferometer represents a minimum and the signal interferometer shows maximum slope in the emission wavelength range of the light source, aimed to achieve maximum sensibility in the measurement.

The signal interferometer is the sensing area where the biochemical interaction happens; therefore, the changes in its surface create a wavelength shift. Meanwhile, the reference interferometer remains constant. As it is represented in Figure 2, ΔIROP (%) can be defined as the subtraction of the IROP (%) value before and after the biochemical accumulation on the sensing surface.

The point-of-care device is based on an optical system and an electromechanic system. The electro-mechanic system permits the sequential measurement of several biosensing cells in a single kit to be carried out. The optical system consists of a laser and a photodiode to carry out the vertical interrogation of both the signal and the reference interferometers, with an incident angle of 10 degrees. The laser spot at the focal distance is around 60 μm in diameter, which allows the measurement of sensing areas with a diameter smaller than 100 μm to be carried out.

### 2.3. Assay Protocol: ELISA Technique and Point-Of-Care (IROP)

#### 2.3.1. ELISA Technique for Interferon Gamma detection

**Figure 3:**
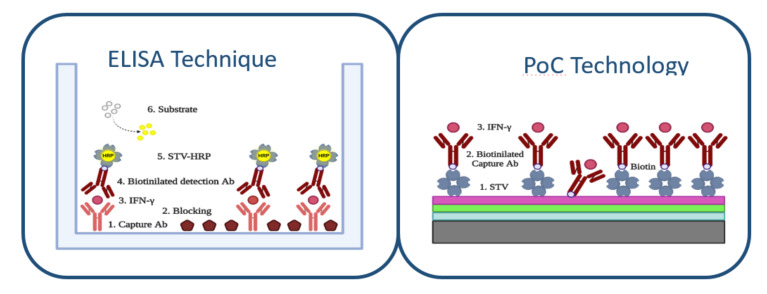
ELISA technique protocol for IFN-γ detection and PoC technology protocol for IFN-γ detection.

The ELISA method was performed by the sandwich type assay (Figure 3). For this purpose, the ELISA plate was immobilized with 50 μL/well of capture antibody antiIFN-γ (from Sigma-Aldrich) during 2 h at 37 °C with a concentration of 5 μg/mL. For cleaning the wells, 5 washing steps of 200 mL/well with PBS-T 0.05% with a microplate washer were done.

For the ELISA technique, due to the size of the well, a blocking step was required. For this purpose, polyvinylpyrrolidone PVP10 (from Sigma-Aldrich) was incubated for 1 h at 37 °C with a 50 μL/well volume and 5 μg/mL concentration. After that, 5 washing steps were done by a microplate washer of 200 μL/well with PBS-T 0.05% (from Sigma-Aldrich).

The recognition molecule IFN-γ was detected through a 90-min incubation, 50 μL/well at 37 °C using the following concentrations 0.025, 0.05, 0.1, 0.2, 0.5, 0.75, 1, 1.5, 2, 2.5, 3, 3.5, 4, 5, and 10 μg/mL. After that, 5 washings of 200 μL/well by a microplate washer of PBS-T 0.05% (from Sigma-Aldrich) were conducted.

Biotinylated secondary antibody antiIFN-γ was incubated for 1 h at 37 °C with a concentration of 5 μg/mL and 50 μL/well of volume and was washed as the previous steps.

Finally, 50 μL in a 0.2 μg/mL concentration of STV labeled with HRP enzyme was deposited on each well for 1 h at 37 °C. Washing steps took place by 5 cycles of PBS-T 0.05% (from Sigma-Aldrich) by a microplates washer. After this, 50 mL of TMB substrate were added and kept in darkness, and were incubated for 30 min. The reaction was stopped with 50 μL of HCl 2N and reading in the ELISA plate reader.

#### 2.3.2. Point of Care Protocol for Interferon Gamma Detection

The sensing surface of nitrocellulose allowed us to biofunctionalize the chip optimally to evaluate the IFN-γ concentration (Figure 3). Firstly, a biofilm of streptavidin STV (from Sigma-Aldrich) was bound to the sensing surface of three Bicells. To optimize the sensing surface in the assay, we evaluated the immobilization of streptavidin with different buffers: sodium carbonate buffer and deionized water buffer. We observed that the immobilization with buffer carbonate was more unstable for our sensing surface of nitrocellulose. The immobilization of streptavidin was carried out by incubation of 50 μg/mL in aqueous buffer with a volume of 4 μL for bicell. The incubation time was 1 h and the temperature was $37{\;}^{{}^{\circ}}$C. After, one washing step with 60 mL of distilled water was done and the chip was dried with clean and dry air. Additionally, to study the stability of the streptavidin monolayer, we studied several washing steps.

Then, a biotinylated antibody antiIFN-γ (from King Fisher) was immobilized over the streptavidin sensing surface. The incubation concentration was 5 μg/mL, with a volume of 4 μL during 1 h at $37{\;}^{{}^{\circ}}$C. The washing step was done with 40 mL of distilled water and dried with clean air.

The strepatividin–biotin interaction [34,35] is the stronger non-covalent biological interaction. This interaction is highly specific, rapid on-rate, and resistant to changes in temperature or pH. The K_d_ of streptavidin-biotin conjugate is 10**^−^**^14^–10**^−^**^15^ M. The streptavidin has four sites of binding for each biotinylated. This complex is highly stable, and the same biotinylated antibody is the blocking agent over the sensing surface.

The sensing surface obtained is specific to the interferon gamma concentration since the whole surface is covered by streptavidin and the antibody itself acts as a blocking agent since it is biotynilated and the biotin has a great affinity for the sensing surface. In addition, to be sure of this specific surface, 10 μg/mL of interferon gamma was incubated for 3 h over the sensing surface of streptavidin, without the biotinylated antibody step, as a negative control.

The interferon gamma detection was made at $37{\;}^{{}^{\circ}}C\;$in a humid environment using concentrations of 0.025, 0.05, 0.1, 0.2, 0.5, 0.75, 1, 1.5, 2, 2.5, 3, 3.5, 4, 5, and 10 μg/mL with an incubation time of 90 min. Finally, a washing step was performed with 20 mL of distilled water and dried with clean air.

To obtain each one of the values of this assay, the measurement of 11 compact chips based on BICELLs was carried out. This resulted in a statistical sample of n = 33. The results obtained are shown as values obtained by repeated measurement + SEM (standard error of the mean).

## 3. Results and Discussion

### 3.1. Optimization of Sensing Surface

**Figure 4:**
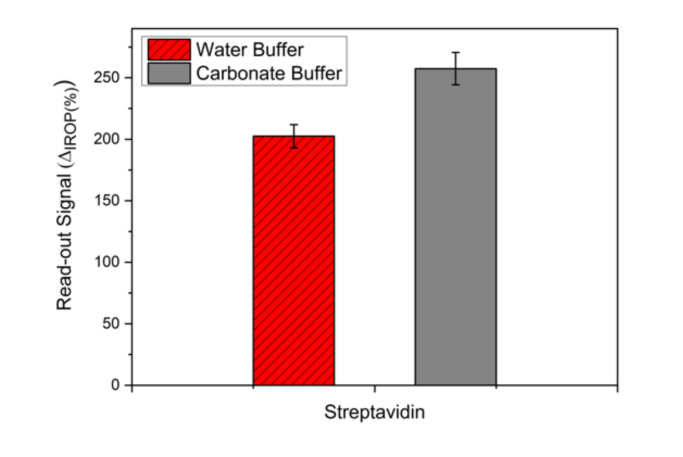
Evaluation of the buffer over the sensing surface: deionized water buffer and carbonate buffer.

**Figure 5:**
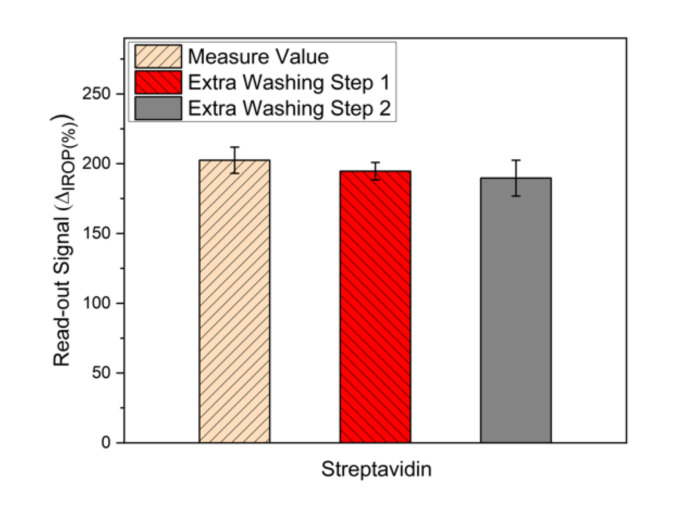
Washing steps of the streptavidin monolayer.

To optimize a monolayer of the streptavidin sensing surface, two buffers were evaluated. In Figure 4, we show the signal obtained for the water buffer and carbonate buffer in the same conditions. It can be seen that the signal for the carbonate buffer is higher than the deionized water buffer. To evaluate the stability of the streptavidin monolayer, we studied consecutive washing steps of 20 mL of distilled water (Figure 5). We can see that the stability of the streptavidin sensing surface immobilized with deionized water buffer is the same, but with the same washing steps, the signal with the carbonate buffer decreased very much and this means that the streptavidin layer was removed.

**Figure 6:**
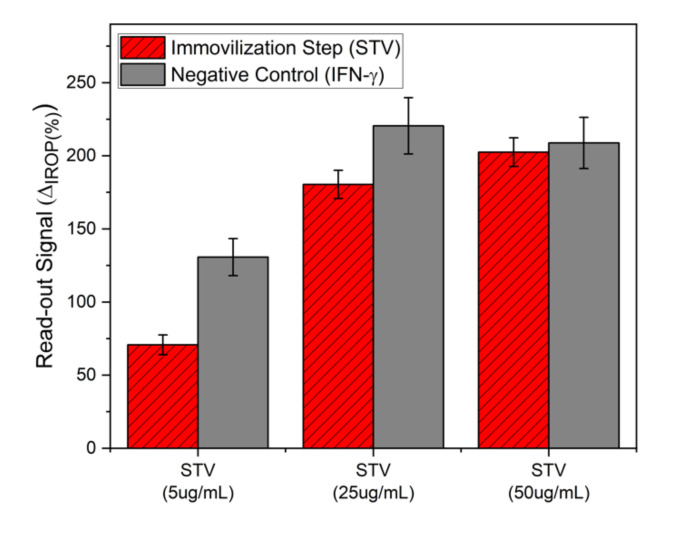
Negative control by immobilization of interferon-gamma over the streptavidin sensing surface.

In addition, the streptavidin dose–response curve was made to ensure surface stability and specificity. This was done by incubation of 5, 25, and 50 μg/mL during 1 h of streptavidin. Then, interferon gamma was incubated at 10 μg/mL for 3 h directly over the streptavidin layer as a negative control. The results are shown in Figure 6. It can be seen that for a streptavidin immobilization concentration of 50 μg/mL, the signal when the incubation of interferon gamma is made is the same and therefore there is no recognition of interferon-gamma over streptavidin.

Finally, the biotinylated antibody was attached to streptavidin by the affinity of streptavidin-biotin. The formed complex by streptavidin and biotinylated antibody antiIFN-γ is stable and therefore, the sensing surface is very specific.

### 3.2. Detection of Interferon-Gamma. Comparison of ELISA and PoC Techniques

**Figure 7:**
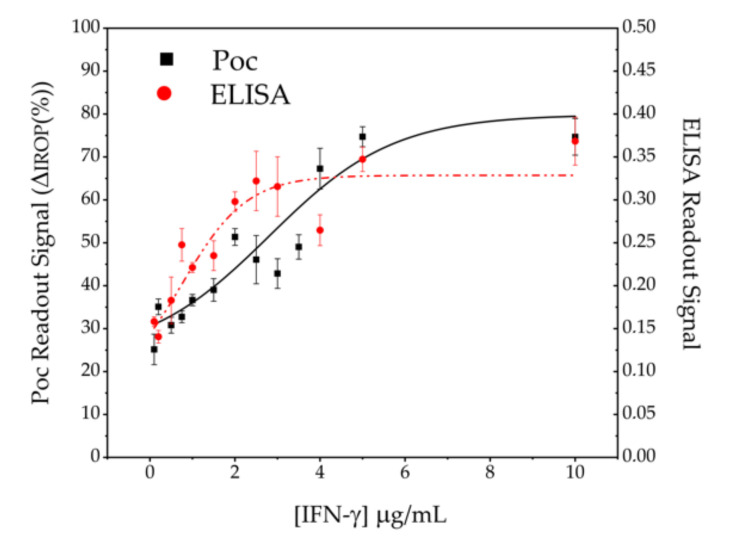
IFN-γ concentration detection. The black squares correspond to the PoC technology and the red dots to the ELISA technique.

According to the conditions of the ELISA that was carried out, it is observed in Figure 7 that the minimum detectable concentration, that is to say, the limit of detection (Lod), under those conditions is 25 ng/mL and that it presents a dynamic range from 25 ng/mL to a value of approximately 200 ng/mL where saturation is reached.

The results obtained with the interferometric optical detection method under the same conditions as the ELISA technique can be seen also in Figure 7. In the PoC technology, label-free tests were carried out and therefore, with a lower number of stages and reagents. The PoC technology presents in the test conditions an Lod of 25 ng/mL and a dynamic range around 400–500 ng/mL higher than in the case of the ELISA technology.

**Figure 8:**
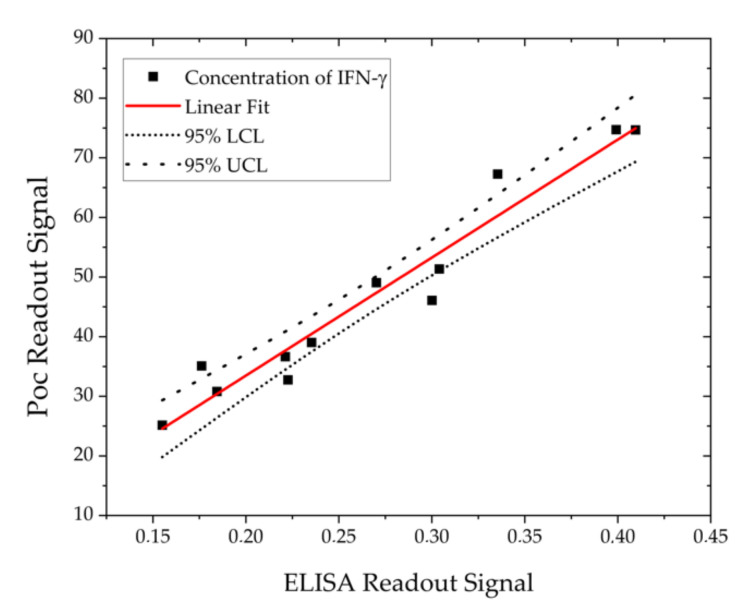
Linear adjustment between the ELISA technique and PoC technology.

Lastly, a comparison between both techniques was made by making a linear adjustment in the lineal range (Figure 8) and it can be seen that it is a good fit and there is agreement between the data obtained by both techniques and it is observed that all the points fall within a linear adjustment with a 95% confidence interval.

## 4. Conclusions

In this work, we used a label-free point-of-care device based on the label-free interferometric optical detection method to evaluate the interferon gamma (IFN-γ) concentration. We used a biosensor with three BICELLs (Biophotonic Sensing Cells) of 100 μm in diameter. The specific sensing surface is reached by the streptavidin-biotin affinity. The biotinylated anti-IFN-γ bioreceptor is bonded to streptavidin and at the same time, due to its small size, is used as a blocking agent on the surface. The point-of-care device can work with a low concentration of reagents and a low sample volume for measurements. In the same conditions, the detection limit of interferon gamma obtained for PoC is the same as that of the ELISA technique (25 ng/mL) using less reagents and steps in the assay. Finally, we showed a good correlation between both techniques in the lineal range studied.
